# Idiopathic Flatfoot in Children and Adolescents Treated with Arthroereisis—Muscle Recession May Not Be Necessary in Feet with Mild Gastrocnemius Shortening

**DOI:** 10.3390/children12091239

**Published:** 2025-09-16

**Authors:** Rebecca Alexandra Jakobs, Harald Böhm, Albert Fujak, Chakravarthy Ugandhar Dussa

**Affiliations:** 1 Department of Trauma and Orthopaedic Surgery, Pediatric and Neuro Orthopaedics, University Hospital Erlangen, Friedrich-Alexander-Universität Erlangen-Nürnberg (FAU), Krankenhausstr. 12, 91054 Erlangen, Germany; rebecca.jakobs@fau.de (R.A.J.); albert.fujak@uk-erlangen.de (A.F.); 2 Gait Laboratory, Orthopaedische Kinderklinik, Bernauerstrasse 18, 83229 Aschau im Chiemgau, Germany; harald.boehm@hawk.de; 3 Department of Orthopaedics and Trauma Surgery, Musculoskeletal University Center Munich (MUM), LMU University Hospital, LMU Munich, Marchioninistraße 15, 81377 Munich, Germany

**Keywords:** Arthroereisis, idiopathic flatfoot, paediatric, flexible flatfoot, De Pellegrin, Screw, Baumann procedure, gastrocnemius recession, subtalar joint, pes planus, gait analysis, oxford foot model, SESA, calcaneus stop procedure

## Abstract

**Highlights:**

**What are the main findings?**

- Idiopathic flexible flatfeet in children and adolescents may be associated with shortening of gastrocnemius muscle.
- Gastrocnemius shortening affects the feet more in the sagittal plane.
- The kinematic deviations in the fore- and hindfoot tend to be more severe in flatfeet with gastrocnemius shortening.
- Gastrocnemius shortening prevents inversion of the hindfoot during push-off.

**What is the implication of the main finding?**

- Arthroereisis is effective in correcting flexible flatfeet, reduces pain, and restores the kinematics of the fore- and hindfoot.
- Following arthroereisis, there is a spontaneous stretching of the calf-muscle especially the gastrocnemius.
- Gastrocnemius recession is not necessary in mild shortening, as the muscle stretches after correction of the flatfoot.

**Abstract:**

**Background:** Arthroereisis is a well-accepted and relatively easy procedure to treat the flexible flatfeet in children and adolescents. A mild calf-muscle shortening is not seldom an accompanying feature. The need for a gastrocnemius recession in addition to arthroereisis is controversial. Therefore, the objective of this study is to investigate the need for a gastrocnemius recession in mild cases of gastrocnemius shortening to improve ankle dorsiflexion in addition to arthroereisis. **Methods:** Twenty-seven patients (ages 9–15 years) who underwent arthroereisis for painful idiopathic flatfeet were included in this non-randomised retrospective study, approved by Friedrich-Alexander University, Erlangen-Nürnberg (22-86-Br). The gait data of 18 typically developed children in same age group was used as reference. Based on the intraoperative Silfverskjöld test, two groups could be identified in the collective, one with shortened of gastrocnemius who underwent gastrocnemius recession (FFGR) and one without (FF). A control group included 18 feet of 18 typically developing children. Outcomes were evaluated by comparing pre- and postoperative clinical assessments including pain scores, gait analysis using a multi-segmental foot model, and radiological imaging. The mean follow-up was 22.1 months, and statistical analysis included a two-factor ANOVA. **Results:** No statistically significant differences in anthropometric, clinical, and gait parameters were observed between the groups preoperatively. Improvements in ankle dorsiflexion and pain were seen in both groups without statistical significance. There was no loss of calf-muscle strength or ankle power. **Conclusions:** Arthroereisis effectively corrects an idiopathic flexible flatfoot and reduces pain in children and adolescents. The gastrocnemius muscle stretches following arthroereisis and therefore, no lengthening is necessary when mildly shortened. The major limitations of this study are its retrospective nature, non-randomisation, and small size of the study collective.

## 1. Introduction

Childhood and adolescent flatfeet are common causes of presentation to paediatric orthopaedic clinics [1,2,3] and is a complex three-dimensional deformity. They are characterised by a flattened or absent medial arch of the foot, forefoot abduction, hindfoot valgus and plantarflexion of the talus [4,5,6,7]. Despite its prevalence of 24% in school children, a standard definition for paediatric flatfoot does not exist in the literature [3,8]. Idiopathic flexible flatfeet account for approximately 95% of cases and may be asymptomatic or symptomatic. While the treatment of both asymptomatic and painful flexible flatfeet remains controversial, rigid deformities accounting for 5% of flatfeet, often require surgical treatment [1,2,3,9,10,11,12,13].

Surgical treatment is warranted in symptomatic flexible flatfeet when conservative treatments fail. Surgical procedures aim at correcting the foot deformity, alleviating pain and improving foot function [7], and improves patient’s quality of life [14,15]. Several surgical procedures have been described to treat a flexible flatfoot: subtalar arthroereisis [10,16], soft tissue procedures [17], osteotomies [18,19] and arthrodesis [20]. Arthroereisis has been increasingly and effectively used to correct an idiopathic flexible flatfoot in children and adolescents. Arthroereisis with a screw, as propagated by De Pellegrin, prevents excessive eversion of the hindfoot [10], while preserving the inversion of the calcaneus and therefore stabilises the subtalar joints (STJs) and midfoot joints.

The incidence of a shortened calf-muscle in idiopathic flatfeet has been reported to be 50–100% [21]. It leads to premature elevation of the heel and increased loading on the forefoot and midfoot joints [22]. This may cause medial or lateral foot pain. The need for calf-muscle lengthening in combination with the flatfoot correction is controversial, with De Pellegrin et al. [10] reporting a 0% and Yontar et al. [10] a 100% need for lengthening. Wang et al. investigated the influence, radiological outcome, and complications following correction of idiopathic flexible flatfeet treated with arthroereisis in combination with and without gastrocnemius recession (GR) [23]. They attributed the satisfactory results rather to the arthroereisis than to GR. The absence of data on clinical preoperative and postoperative ankle dorsiflexion in some studies may fail to identify the need for calf-muscle lengthening.

Therefore, the purpose of this study was to evaluate need for the lengthening of gastrocnemius recession in mild shortening of the gastrocnemius muscle. Our hypotheses were (a) arthroereisis effectively corrects the flexible idiopathic flatfoot, reduces pain, improves the clinical, radiological, and foot kinematics and (b) mild gastrocnemius shortening in paediatric flatfoot following arthroereisis does not need a GR surgery and lengthens spontaneously during follow-up.

## 2. Patients/Materials and Methods

A retrospective non-randomised study was conducted on children and adolescents between 9 and 15 years with idiopathic flatfeet treated with arthroereisis between 01/2012 and 12/2021 at the Orthopaedic Hospital for Children, Aschau. The study was approved by the ethics committee of the Friedrich-Alexander University, Erlangen-Nürnberg (22-86-Br). The study consisted of 2 study groups and a control group consisting of 18 typically developed children between the age 9 and 15. Inclusion criteria were children with painful idiopathic flatfoot following a failed orthotic treatment, presence of pre- and postoperative clinical, radiological, and 3-dimensional instrumented gait data and pain scores. The exclusion criteria were rigid flatfeet, tarsal coalitions, feet needing additional osteotomies of foot and ankle with arthroereisis, severe obesity [24], arthroereisis using other implants, and children with severe foot pain preventing an instrumented gait analysis.

### 2.1. Clinical Assessment

The following parameters were extracted from a standardised preoperative and follow-up clinical examination protocol: passive ankle dorsiflexion (0° knee flexion and 90° knee flexion) and plantarflexion (angle between the hindfoot and the leg) measured using a hand-held goniometer, ankle plantarflexion strength and number of heel-raises. In addition, the children and adolescents were asked to rate “pain in the medial or lateral side of the foot at its worst” from 0 to 10 as in the German version of the Foot Function Index Questionnaire [25].

### 2.2. Radiological Assessment

Standardised lateral and anterior–posterior (AP) radiographs of the foot were taken under full weight-bearing [26]. The radiographs were measured by a single trained person as described by Davids using the PACS software^®^ (Allgeier Medical IT GmbH Freiburg Germany, version 8.3.1). Five parameters on the AP view and seven on the lateral view were selected based on a previous study on flatfeet [27].

### 2.3. Gait Analysis

Children were asked to walk barefoot at a self-selected speed along a 13 m walkway with embedded force plates (AMTI AG, Watertown, MA, USA), which was repeated until 6 clean steps were obtained on the force plates of each foot. A marker-based 8-camera system (Vicon Motion Systems^®^, Oxford, UK) was used to measure foot kinematics according to the Oxford Foot Model (OFM). The gait analysis data of 18 typically developed children between the age 9 and 15 (mean 11.9 years) was used as reference.

### 2.4. Surgical Technique and Indication for Gastrocnemius Recession

Arthroereisis was performed under general anaesthesia with a 6.5 mm non-cannulated cancellous screw as described by De Pellegrin [10]. On-table ankle dorsiflexion was checked in knee flexion and extension (Silfverskjöld test) [28,29]. A passive ankle dorsiflexion of ≥10° with knee in extension was considered as normal. A single-level gastrocnemius aponeurotomy (Baumann type) was performed if the passive ankle dorsiflexion was <10°. All patients were allowed to walk full weight-bearing with a below-knee cast for 6 weeks and then followed by an ankle–foot orthosis (AFO) for further 3 months.

### 2.5. Statistical Analysis

We identified 2 groups in our study collective; those who received a GR (FFGR) and those who did not (FF). A 2-factor ANOVA (FFGR and FF) with repeated measures on time (pre-and post-surgery) was performed to determine the significance of the data using MatLab^®^ (MatLab statistics toolbox 5.3, The Mathworks). The significance level (*p*) for the clinical parameters was set to *p* < 0.05. Bonferroni correction was used on the same dataset accounting for multiple testing. The significance level *p* was corrected to <0.007 for the lateral radiographs (7 parameters), *p* < 0.01 for the anterior–posterior radiographs (5 parameters) and *p* < 0.005 for the 11 gait parameters. A post hoc power analysis for the two most relevant parameters associated with GR, the Naviculo-cuboid overlap and the peak midfoot dorsiflexion during stance-phase of gait, was conducted using the software package, G*Power 3.1.9.2 [30].

## 3. Results

Forty-four feet in 27 children with a mean age of 11.9 years (range, 9–15 years) were included in the study. In total, 18 typically developing children, contributing 18 feet, were included as the control group. The mean follow-up time was 22.1 months. The anthropometric and clinical data including pain assessment are presented in Table 1. Group FFGR consisted of 21 feet in 13 children with a mean age of 12.4 ± 1.6 years, and Group FF consisted of 23 feet in 14 children with a mean age of 12.1 ± 1.2. The pre- and postoperative data showed no statistical significance between groups. The clinical examination in the gait laboratory showed a difference in the passive dorsiflexion with extended knee between groups (2.8° ± 5.4° vs. 4.4° ± 4.0°) and a greater difference with the Silfverskjöld test between groups indicating a more tightness of gastrocnemius in the FFGR than in the FF group. There was a discrepancy as to the measured passive dorsal extension in the gait laboratory and on the surgical table. At follow-up, the passive ankle dorsiflexion significantly increased in both groups and foot pain significantly decreased in both groups.

The radiological parameters are presented in Table 2. Preoperatively, three out of seven parameters on the lateral radiographs were significantly impaired in the FFGR group. Following surgery, all measured radiological parameters showed statistically significant improvements in both groups.

Table 3 displays the mean spatio-temporal parameters, foot kinematics, ankle moments, and powers for both the study groups and TD controls. There were no significant differences in velocity, step length, and cadence between the study groups preoperatively.

**Figure 1:**
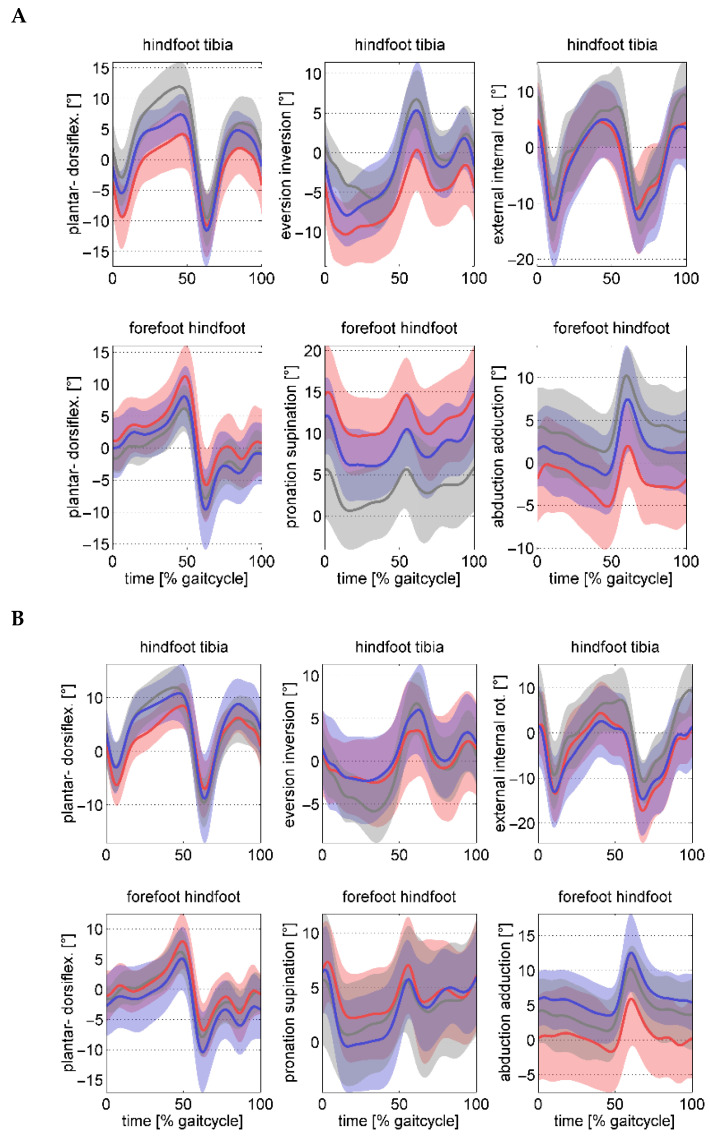
Foot kinematic waveforms preoperatively (**A**) and postoperatively (**B**) across the gait cycle. The FF group is represented in blue, the FFGR group in red, and the TD controls in grey. The shaded area indicates one standard deviation from the mean waveform.

**Figure 2:**
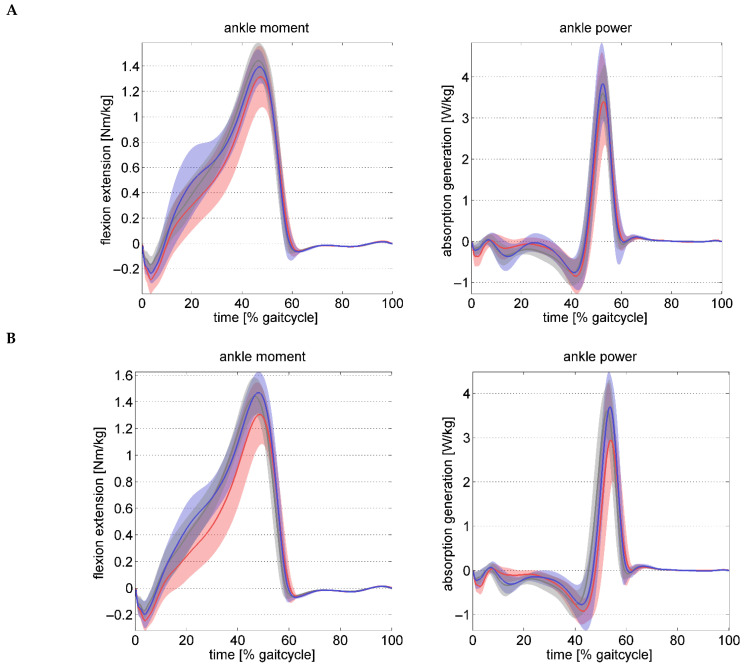
Ankle kinetic waveforms preoperatively (**A**) and postoperatively (**B**) across the gait cycle. The FF group is represented in blue, the FFGR group in red, and the TD controls in grey. The shaded area indicates one standard deviation from the mean waveform.

Figure 1 shows pre- and postoperative the kinematic data and Figure 2, the kinetic waveforms for both groups over the gait cycle. The preoperative hindfoot kinematics relative to the tibia, did not show any significant differences in the peak ankle dorsiflexion in stance. Although the hindfoot inversion at take-off was not significant (0.007) between groups, only the hindfoot in the FFGR group remained in eversion at take-off. The hindfoot in the FF group inverted at take-off. At follow-up, both parameters significantly improved in the FFGR group with no difference to those in the FF group. Except for the forefoot abduction, the preoperative kinematics of the forefoot with respect to the hindfoot were not significantly different, although the deviations were pronounced in the FFGR group in all three planes (Table 3).

The peak ankle moment was slightly but significantly reduced in the FFGR group preoperatively. At follow-up, the ankle moment and power reduced to a similar extent in both the study groups without significant difference between groups.

## 4. Discussion

The results of the study support the first hypothesis that the arthroereisis of the STJ effectively corrects the flexible idiopathic flatfoot and significantly reduced pain and improved clinical, radiographic, and foot kinematic parameters. The improvements in all the above parameters were found in both groups, without significant differences, suggesting that the arthroereisis alone was responsible for the improvement. The second hypothesis was also true. GR is not necessary for mild gastrocnemius shortening in paediatric flatfoot following arthroereisis. The gastrocnemius stretches spontaneously during follow-up. There were no significant differences in the ankle dorsiflexion, pain scores, and foot kinematics between groups.

Arthroereisis is a widely used minimally invasive procedure with low risk and can be combined with forefoot and hindfoot osteotomies [9]. Several papers support the effectiveness of this procedure alone in restoring the longitudinal arch and in alleviating symptoms [4,6,9,10,14,15,16,31]. Reduced surgical time, costs, morbidity, and immediate mobilisation make this procedure attractive [17].

Chong et al. in a prospective study comparing arthroereisis to lateral column lengthening concluded in favour of arthroereisis for the treatment of painful flatfoot in children [32]. At the 1-year follow-up, significant improvements in radiographic, pedobarographic, and kinematic measurements of the hindfoot and midfoot were reported in both groups. However, there was no mention of calf-muscle shortening in their collective. Although the FFGR group had pronounced deviations in foot kinematics preoperatively, the differences in preoperative clinical passive ankle dorsiflexion measured in the gait laboratory between the FF and FFGR groups was <2° with straight knee and 2° with flexed knee. Such mild differences do not justify a GR. Although the improvements in the ankle dorsiflexion measured clinically and during gait were greater in the FFGR group at the 2-year follow-up, it is not surprising that they were not different between the FFGR and FF groups. The effectiveness of arthroereisis in the restoration of foot kinematics could be proven with a multi-segmental foot model in that, the kinematic parameters in both groups were comparable to those of the TD group. A stretching effect of the calf-muscle can be expected following arthroereisis due to the stabilisation of the subtalar joint.

Interestingly, despite insignificant differences in the preoperative passive ankle dorsiflexion between FFGR and FF groups, significant differences in three radiological parameters (reduced calcaneal pitch and first metatarsal declination angle and increased overlapping between the navicular and cuboid bones) were seen between groups. The feet in the FFGR group were radiologically worse than those in the FF group. In the absence of significantly deranged inter-segmental foot kinematics, it would be difficult to determine why the feet in the FFGR group were radiologically severely affected than those in the FF group. Another reason could the absence of correlation between foot function and radiographic observation [27]. The set low significance level of <0.005 for foot kinematics in contrast to 0.01 for AP and 0.007 for lateral radiographs could be another reason.

The role of the shortened gastrocnemius in an idiopathic flexible flatfoot deformity and the importance of its treatment is not very clear in the literature. A minimum of 10° ankle dorsiflexion is necessary for normal stance-phase ankle kinematics during gait [14]. Insufficient ankle dorsiflexion leads to premature heel-rise and persistent eversion or failure of inversion of the hindfoot in the mid and terminal stance-phase. Absence of locking of the subtalar joint as a result the midtarsal joints causes excessive dorsiflexion of the forefoot (midfoot break). In addition, there is an increased supination and abduction of the forefoot. All these three deviations in the forefoot although not significant, were seen in the FFGR group. The loss of lever-arm due to midfoot break leads to reduced ankle moment and premature loss of ankle power as evident in Table 3.

The Baumann procedure (GR) improves ankle dorsiflexion in the presence of a shortened gastrocnemius muscle [29]. Herzenberg et al. showed an 8° improvement following a single-level GR in ankle dorsiflexion with knee in extension in a cadaver study. Adding a second gastrocnemius recession improved the ankle dorsiflexion by a further 6° [29]. In the present study, a single-level GR was performed in all children in the group FFGR and the passive ankle dorsiflexion improved by 6°. It remains unknown as to why some flatfeet present with shortening of the calf-muscle while others do not. The greater deviation of the kinematic and radiological parameters in the FFGR group from the norm suggests a pathological influence of the gastrocnemius muscle. Spontaneous improvements in the ankle dorsiflexion at follow-up seen in the FF group without GR suggest stretching of gastrocnemius following arthroereisis and therefore, does not justify a GR. However, this question needs to be further pursued by randomised controlled studies.

On the other hand, studies from Li et al. [17], and Jay and Din [14] justify the need for calf-muscle lengthening following arthroereisis. Li et al., using two different implants, investigated the outcomes of subtalar arthroereisis combined with Achilles tendon or gastrocnemius recession and medial soft tissue tightening for treating severe flexible flatfoot deformities using pre- and postoperative radiological parameters, AOFAS score, and VAS score [17]. However, they did not have any control group in which no calf-muscle lengthening was performed. They described gastrocnemius tightness as an “essential cause of the arch collapse” and concluded that, the combination of subtalar arthroereisis and calf-muscle lengthening leads to an effective correction of flexible flatfoot [17]. A retrospective study from Jay and Din, showed a significant improvement in AOFAS, Ankle–Hindfoot Scale and subjective assessments of pain, function, show wear, and overall satisfaction, clinical outcomes, and the children’s overall health following arthroereisis and gastrocnemius recession [14]. None of the above two studies used a gait analysis for justification, nor did they mention the threshold value for the need for calf-muscle lengthening. Gait analysis using a three-dimensional multi-segmental foot model and the measurement of calf-muscle strength and number of heel-raises, as performed in this present study, in our opinion, gives more objective and reliable information to the foot function than functional outcome scores, which are subjective in nature.

The drawbacks of the present study are the small number of patients and the retrospective nature of the study. Therefore, the results of this study should be interpreted in light of the above limitations. The post hoc power analysis revealed for the surgical effect on the Naviculo-cuboid overlap an effect size of 0.33, requiring 58 participants to achieve a power of 80%. Similarly, the peak hindfoot–tibia dorsiflexion with an effect size of 0.32 requires the inclusion of 62 participants to achieve a power of 80%. Considering the seldomness of occurrence of pain and the need for surgery (<5%) for a paediatric flexible flatfoot, it may not be feasible to include these numbers of patients in each group. Despite these two drawbacks, the data in the present study suggests that the shortening of the gastrocnemius influences the kinematics of the hind- and forefoot.

To conclude, arthroereisis affectively corrects the flexible flatfoot and can reduce pain as well as restore the radiological and kinematic parameters. A GR is not necessary in cases of mild calf-muscle shortening, as passive dorsiflexion improves due to the stretching of the calf-muscle. Given the limitations of our retrospective study, future randomised controlled trials are needed to further validate our findings.

## Figures and Tables

**Table 1:** Mean values and standard deviations in anthropometric and clinically tested data for both groups are presented pre- and postoperatively, compared with typically developed (TD) controls. Significant results for clinical tests are shown in bold, with *p* < 0.05.

	FFGR		FF		TD	ANOVA *p*-Values		
Parameter	Pre	Post	Pre	Post		Group	Surgery	Interaction
**Anthropometry**								
No. of subjects/feet	13/21		14/24		18/18			
Gender (m/f)	7/14	7/14	12/12	12/12	8/10			
Age (years)	12.4 (1.6)	14.4 (1.7)	12.2 (1.2)	14.3 (2.1)	12.1 (2.6)	0.632	**<0.001**	0.844
Height	155 (10)	165 (9)	159 (8)	169 (10)	155 (17)	0.175	**<0.001**	0.752
Weight (kg)	48.4 (8.8)	60.2 (12.0)	50.0 (11.1)	60.3 (14.1)	43.5 (14.5)	0.812	**<0.001**	0.396
$\mathrm{BMI}\;(\mathrm{kg}/m^{2}$)	20.2 (3.7)	22.0 (3.5)	19.7 (3.4)	20.9 (3.3)	17.6 (2.6)	0.437	**<0.001**	0.109
**Clinical Assessment**								
Passive ankle dorsiflexion [°] (0° knee flexion)	2.9 (5.4)	8.9 (6.2)	4.4 (4.0)	10.2 (7.4)	17.9 (7.1)	0.348	**<0.001**	0.910
Passive ankle dorsiflexion [°] (90° knee flexion)	10.5 (8.2)	14.5 (5.3)	9.8 (5.6)	15.0 (6.4)	23.8 (8.4)	0.961	**<0.001**	0.570
Silferskjöld test [°]	7.6 (3.7)	5.6 (4.0)	5.4 (4.9)	4.8 (3.8)	5.9 (5.7)	0.115	0.111	0.391
Passive ankle plantarflexion [°]	37.4 (7.4)	31.9 (10.7)	36.7 (8.8)	32.5 (7.5)	39.4 (8.3)	0.978	**0.002**	0.664
Ankle plantarflex. strength (1–5)	4.9 (0.3)	4.9 (0.3)	4.9 (0.3)	4.9 (0.5)	5.0 (0.0)	0.820	0.892	0.618
Heel-raises (o.)	15.1 (5.4)	15.3 (5.2)	17.3 (4.6)	17.5 (5.7)	19.4 (1.6)	0.100	0.838	0.989
Foot pain VAS [0, 10]	3.5 (2.6)	2.5 (2.6)	4.2 (2.3)	1.8 (2.9)	0 (0)	0.973	**0.001**	0.159

**Table 2:** Mean values and standard deviations in radiographic data for both groups are presented pre- and postoperatively. For the TD group, radiographs were not available, instead, available reference values from children [29] were used for comparison. Significant results are shown in bold: lateral radiograph *p* < 0.007 (Bonferroni corrected) and anterior–posterior *p* < 0.01 (Bonferroni corrected).

	FFGR		FF		TD	ANOVA *p*-Values		
Parameter	Pre	Post	Pre	Post		Group	Surgery	Interaction
**Lateral Radiographs**								
Talo-calcaneal [°]	43.2 (10.4)	37.9 (12.5)	48.8 (10.5)	38.7 (11.2)	49 (7)	0.313	**<0.001**	0.064
Talo-1 Metatarsal [°]	21.4 (6.4)	9.7 (9.9)	18.3 (10.5)	3.9 (8.3)	13 (8)	0.068	**<0.001**	0.307
Calcaneal pitch [°]	8.7 (6.4)	11.8 (5.6)	14.1 (4.3)	16.7 (5.1)	17 (6)	**0.002**	**<0.001**	0.628
Talonavicular [°]	6.4 (8.7)	−0.5 (10.2)	4.7 (10.6)	−6.1 (8.3)	N/A	0.172	**<0.001**	0.095
Naviculocuneiforme [°]	20.1 (6.2)	16.5 (7.6)	18.3 (6.0)	13.4 (5.2)	N/A	0.146	**<0.001**	0.510
Metatarsal-1 declination [°]	−13.1 (3.1)	−16.4 (3.4)	−16.3 (3.1)	−18.1 (2.6)	N/A	**0.003**	**<0.001**	0.112
Naviculo-cuboid overlap (%)	85.0 (7.5)	69.8 (10.6)	80.3 (9.8)	62.3 (9.5)	47 (14)	**0.004**	**0.001**	0.478
**Anterior–Posterior Radiographs**								
Talo-calcaneal angle [°]	20.9 (7.1)	13.6 (8.8)	22.2 (10.8)	13.6 (7.3)	N/A	0.759	**<0.001**	0.688
Talonavicular coverage [%]	24.1 (11.0)	7.7 (10.8)	23.1 (11.8)	2.4 (7.8)	20 (10)	0.218	**<0.001**	0.267
Talo-1 metatarsal [°]	12.0 (6.3)	0.4 (10.0)	12.1 (12.1)	−1.4 (7.7)	10 (7)	0.724	**<0.001**	0.574
Talo-2 metatarsal 2 [°]	23.0 (7.5)	11.0 (11.0)	21.9 (11.7)	6.9 (8.1)	N/A	0.304	**<0.001**	0.371
Calcaneal-4 metatarsal [°]	8.2 (6.7)	5.5 (6.3)	5.5 (7.2)	1.7 (6.6)	N/A	0.086	**<0.001**	0.498

**Table 3:** Mean values and standard deviations in gait analysis data for both groups are presented pre- and postoperatively, compared with TD controls. Significant results for gait analysis are shown in bold, with *p* < 0.005 (Bonferroni corrected).

	FFGR		FF		TD	ANOVA *p*-Values		
Parameter	Pre	Post	Pre	Post		Group	Surgery	Interaction
**Spatio-temporal [non-dimensional units]**								
Velocity	45.4 (7.6)	43.2 (4.1)	45.6 (5.0)	44.6 (5.3)	47.0 (5.5)	0.583	0.035	0.452
Step length	78.9 (9.1)	75.2 (5.7)	78.4 (5.3)	76.5 (6.7)	79.1 (8.0)	0.819	**0.003**	0.336
Cadence	58.1 (5.5)	58.1 (2.7)	59.0 (4.2)	58.5 (3.8)	59.6 (3.2)	0.559	0.598	0.611
**Tibia-hindfoot kinematics [°]**								
Peak dorsiflexion stance	4.9 (5.0)	9.7 (3.8)	8.4 (3.0)	11.6 (5.0)	12.7 (5.0)	0.014	**<0.001**	0.243
Peak ankle eversion stance	−11.5 (4.0)	−3.9 (4.7)	−8.6 (3.8)	−4.1 (4.8)	−7.3 (2.9)	0.158	**<0.001**	0.082
Ankle inversion at take-off	−0.0 (5.0)	3.7 (5.6)	4.6 (5.3)	5.9 (5.0)	7.3 (3.9)	0.007	0.015	0.221
**Hindfoot–forefoot kinematics [°]**								
Peak midfoot dorsiflexion stance	11.9 (4.8)	8.6 (4.4)	8.6 (4.6)	5.6 (4.9)	6.7 (3.2)	0.008	**0.001**	0.845
Peak midfoot supination stance	16.9 (4.7)	9.0 (3.4)	13.2 (4.4)	8.4 (4.4)	7.3 (5.3)	0.023	**<0.001**	0.080
Peak midfoot abduction stance	−6.0 (4.9)	−2.5 (6.0)	−2.4 (4.8)	1.9 (5.2)	0.9 (4.3)	0.007	**<0.001**	0.581
**Ankle kinetics**								
Peak ankle moment stance [Nm/kg]	1.34 (0.23)	1.34 (0.22)	1.42 (0.12)	1.49 (0.16)	1.47 (0.15)	0.020	0.209	0.190
Peak ankle power stance [W/kg]	3.95 (1.10)	3.56 (1.03)	4.38 (0.77)	4.20 (0.94)	4.21 (0.47)	0.044	0.029	0.392

## Data Availability

On request.
